# Single‐cell RNA sequencing in cancer research

**DOI:** 10.1186/s13046-021-01874-1

**Published:** 2021-03-01

**Authors:** Yijie Zhang, Dan Wang, Miao Peng, Le Tang, Jiawei Ouyang, Fang Xiong, Can Guo, Yanyan Tang, Yujuan Zhou, Qianjin Liao, Xu Wu, Hui Wang, Jianjun Yu, Yong Li, Xiaoling Li, Guiyuan Li, Zhaoyang Zeng, Yixin Tan, Wei Xiong

**Affiliations:** 1 NHC Key Laboratory of Carcinogenesis and Hunan Key Laboratory of Cancer Metabolism, The Affiliated Cancer Hospital of Xiangya School of Medicine, Hunan Cancer Hospital, Central South University, Changsha, Hunan China; 2 Key Laboratory of Carcinogenesis and Cancer Invasion of the Chinese Ministry of Education, Cancer Research Institute, Central South University, Changsha, Hunan China; 3 Hunan Key Laboratory of Nonresolving Inflammation and Cancer, Disease Genome Research Center, the Third Xiangya Hospital, Central South University, Changsha, Hunan China; 4 Department of Medicine, Dan L Duncan Comprehensive Cancer Center, Baylor College of Medicine, Houston, Texas USA; 5 Department of Dermatology, the Second Xiangya Hospital, Central South University, Changsha, Hunan China

**Keywords:** Single‐cell RNA sequencing, Tumor heterogeneity, Invasion and metastasis, Immune escape, Tumor microenvironment

## Abstract

Single-cell RNA sequencing (scRNA-seq), a technology that analyzes transcriptomes of complex tissues at single-cell levels, can identify differential gene expression and epigenetic factors caused by mutations in unicellular genomes, as well as new cell-specific markers and cell types. scRNA-seq plays an important role in various aspects of tumor research. It reveals the heterogeneity of tumor cells and monitors the progress of tumor development, thereby preventing further cellular deterioration. Furthermore, the transcriptome analysis of immune cells in tumor tissue can be used to classify immune cells, their immune escape mechanisms and drug resistance mechanisms, and to develop effective clinical targeted therapies combined with immunotherapy. Moreover, this method enables the study of intercellular communication and the interaction of tumor cells and non-malignant cells to reveal their role in carcinogenesis. scRNA-seq provides new technical means for further development of tumor research and is expected to make significant breakthroughs in this field. This review focuses on the principles of scRNA-seq, with an emphasis on the application of scRNA-seq in tumor heterogeneity, pathogenesis, and treatment.

## Background

Malignant tumors are caused by genetic mutations that result from the influence of endogenous and environmental factors, and this challenging aspect of oncology has always been a hot topic in medical research [1, 2]. Tumor development is a complex and multi-stage process whereby normal cells develop into malignant tumors, through a series of multiple gene mutations and accumulation in somatic cells. In normal somatic cells due to several factors, genomic instability among them, mutations replicate and accumulate by repeated proliferation and division processes, with important gene mutations resulting in changes in cell phenotypes. During the development of a variety of tumors, several important gene mutations are common which drive the malignant differentiation of cells, as seen by the limitless proliferation, metastasis, and angiogenesis [3–5]. Driver mutation is a key molecular event in tumor occurrence, which affects the degree of malignancy and the prognosis of patients [6]. However, after various divisions and proliferation throughout the process of cell differentiation, the numerous biological or genetic differences within the tumor cells result in the formation of complex tumor heterogeneity [7]. Additionally, tumor tissues differentiate into different cell types and subsets, and develop multiple resistance and proliferation advantages depending on their microenvironments [8–10]. Tumor heterogeneity is the main driving force of drug resistance [11].

Tumor microenvironments play an important role in tumor development and heterogeneity [12]. It is the microenvironmental selection force that may determine the optimal phenotypic properties, that is, the cellular characteristics resulting in the greatest fitness [13]. This is seen by tumor cells at the tumor-host interface which exhibit features that promote invasion and metastasis, whereas cells inside the tumor tissue maximize proliferation by promoting metabolism, such as angiogenesis [14, 15]. In tumor microenvironments, except in malignant cells, the composition and the infiltration degree of immune cells in different tumor types are different [16]. When more T cells infiltrate the tumor tissue, the volume of tumor tissues remains smaller [17] and the patients have a better prognosis [18]. At the same time, various other components of tumor tissues, such as macrophages and neutrophils [19], also closely regulate the immune microenvironment of the tumor. Thus, the sensitivity of different individuals to immunotherapy has an extensive heterogeneity [20, 21]. Additionally, all cell types within the tumor microenvironment interact with each other through cellular communication mechanisms, which increase the complexity of tumor development. Understanding these communication processes between tumor cells are crucial for the formulation of effective anticancer immunotherapy strategies [22–24].

Tumor heterogeneity plays an important role in cancer progression and it is particularly important to thoroughly understand the gene expression patterns of individual cells [25–27]. Common sequencing methods combine thousands of multiple subsets cells for sequencing. So the rare cell clones, which may play an important role in tumor progression, are covered up. It is impossible to accurately track individual cell mutations in the process of tumor progression [28]. Next-generation sequencing (NGS) methods can be used to assess tumor heterogeneity, to track changes within the heterogeneity, and to assess the selective evolution of tumor cells during the course of treatment [11]. Single-cell RNA sequencing (scRNA-seq) overcomes the limitations of traditional RNA sequencing methods, by measuring the whole transcriptome at a single-cell resolution and distinguishing different cell types in tumor tissue. Moreover, this enables a clearer understanding of the molecular mechanisms promoting tumor occurrence, and reveals the somatic mutations throughout the course of tumor evolution [29]. scRNA-seq of tumors at different time points can identify key gene mutations, as well as the dynamic change of the tumor heterogeneity over time. Additionally, this method enables the monitoring of rare cell mutations during processes of tumor occurrence and development, such as the acquisition of invasive and metastatic abilities, as well as the infiltration and activation of immune cells and other important processes [30]. scRNA-seq can also be combined with immune checkpoint therapy to specifically detect the transcriptional activity of immune checkpoints, or to specifically screen neoantigens with high transcription levels [31, 32]. Research on scRNA-seq technologies have seen a significant increase in the last few years, thereby providing new opportunities and strategic approaches for the clinical treatment of cancer. With the development of sequencing technology, the sensitivity and accuracy of detection are gradually improving, and the cost is gradually reducing; thus scRNA-seq has become an important technical tool in tumor research. This review focuses on scRNA-seq, with emphasis on the application of scRNA-seq in tumor heterogeneity, pathogenesis, and treatment.

## Overview of scRNA-seq

It is not possible to sequence RNA directly from single cells. Therefore, scRNA-seq must first convert RNA into cDNA and amplify it by polymerase chain reaction (PCR) or by *in vitro* transcription (IVT) before subsequent sequencing [33]. There are two main problems with this process: first, the loss of RNA must be minimized during reverse transcription; second, amplification should produce enough DNA for sequencing and control the impact of non-single-cell noise [34]. To address these shortcomings, several generations of scRNA-seq technologies are being innovated and improved to adapt to the expanding research scope. scRNA-seq technology has unique advantages and applicable detection content.

**Fig. 1:**
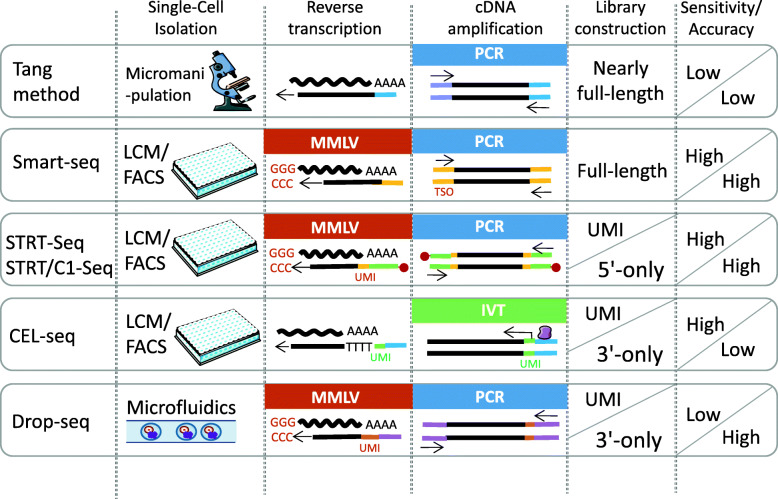
Schematic overview of five scRNA-seq methods Summary of the Tang method, Smart-seq, and the UMI-based sequencing methods STRT-seq, CEL-seq, Drop-seq. Comparative differences of the processes of these methods are outlined: scRNA-seq, reverse transcription, cDNA amplification, purifying and filtration, and library construction. Tang method is the earliest scRNA-seq technology. Single cells are separated by micromanipulation. The overall sequencing sensitivity and accuracy are relative low. In Smart-seq, RNA is reverse transcribed by Moloney mouse leukemia virus(MMLV). The sequencing range can reach the full-length cDNA. It has higher sensitivity and accuracy. STRT-seq and STRT/C1-seq introduce UMI on the basis of Smart-seq and labele with biotin at the 5 ‘end, which can be recovered by magnetic beads. This sequencing method improves the sensitivity and accuracy, but has a strong 5’ end bias. CEL-seq obtains 3 ‘terminal fragment by IVT. The sequencing sensitivity is high, but there is a strong 3’ end bias and the accuracy is low. Drop-seq uses microfluidic technology to package a single cell into an independent droplet, which greatly increases the capture capacity and library capacity of single cell. It has great advantages in detecting a large number of single cell sequencing samples, but the sequencing sensitivity is low

Generally, the scRNA-seq consists of four steps:(1) isolation of single cells, (2) reverse transcription, (3) cDNA amplification, and (4) sequencing library construction [34](Fig. 1). Isolation of single cells mainly includes cell selection, random seeding/dilution, laser microdissection (LCM), fluorescence-activated cell sorting (FACS), and microfluidic/microplate methodology [35, 36]. FACS is the most commonly used method. Manual cell selection is used during the early stage [37], however, the isolation efficiency is low. Microfluidic technology is applied in Drop-seq to wrap a single-cell into an independent microdroplet, which includes oligonucleotide primers, unique molecular identifiers (UMI), DNA bases and cells(Fig. 1). Microfluidic technology considerably increases the single-cell catch and library capacity, thereby enabling thousands of cells to be analyzed simultaneously; therefore, highlighting a great advantage of this method to screen large numbers of cells for sequencing [38, 39].

Reverse transcription and cDNA amplification are important steps to ensure increased sensitivity and accuracy by scRNA-seq. In the reverse transcription process, most methods use oligodT primers, but this also leads to the exclusion of long non-coding RNA (lncRNA), circular RNA, and other non-coding RNA. From the different methods of reverse transcription and amplification, scRNA-seq can be roughly divided into three categories: addition of poly(A) to RNA followed by PCR, IVT, and Moloney murine leukemia virus template switching method. As Fig. 1 shows, in the Tang method, poly(A) was added at the 3’-end of RNA and amplified by PCR. This method can be used to amplify almost the full length of the transcript; therefore, this method potentially finds many neglected new transcripts, and estimates their abundance in cells by the frequency of their occurrence in the mRNA sequence [40]. However, a disadvantage of this method is that it has a 3′ bias and the low efficiency of enzymatic reaction leads to the decrease of sequencing sensitivity and loss of low-expression transcription, so it is not commonly used [41]. IVT uses a composite primer carrying a T7 promoter sequence at the 5’ end. After the second strand cDNA synthesis, the samples are pooled and subjected to an IVT reaction. However, because this method begins with transcription from one end of the sample RNA, there is a large 3′ bias compared to that observed in PCR. This method is applied in CEL-seq and CEL-seq2 for the amplification step [42, 43](Fig. 1). The third method is referred to as template switching (TS) where cell lysates or total RNA are reverse transcribed by Moloney Mouse Leukemia Virus (MMLV). When the reverse transcription reaction reaches the 5′ end of RNA molecule, the terminal transferase activity of MMLV increases some non-template nucleotides at the 3′ end of cDNA. These extra nucleotides work as a docking site for a helper oligonucleotide (template switching oligonucleotide, TSO) that carries three riboguanosines at its 3′ end. The reverse transcriptase is then able to convert from mRNA to the DNA of the TSO and synthesize a cDNA strand using the helper oligonucleotide as a template. The full-length cDNA synthesized by this method contains the complete 5′ end of mRNA concurrently reduces the 3´ bias of transcripts. This method is applied in Smart-seq [44, 45], Smart-seq2 [46, 47], and STRT-seq [48](Fig. 1).

Prior to sequencing, it is important to multiplex samples to increase throughput. A single cell is labeled with a unique barcode, so cells from different samples can be pooled together without confusion. Hence, researchers can analyze multiple samples together on the same sequencing lane. The application of barcode enables the samples to be combined before sequencing at the early stage of the experiment; thus, a single round of amplification is sufficient, which considerably reduces the quantity of reagents needed and the workload required for subsequent sequencing [49]. In the process of scRNA-seq and analysis, in order to avoid the PCR bias, a 6-8-base-pair (bp) barcode unique molecular identifier (UMI) is introduced into scRNA-seq. UMI stands for the identity of the cDNA and barcodes each individual mRNA molecule within a cell during reverse transcription. Sequenced reads that arise from PCR-duplicated tags will have the same UMI, which effectively reduce PCR bias [49].CEL-seq [50], CEL-seq2 [43], STRT-seq, STRT/C1-seq, and Drop-seq are all tag-based sequencing methods(Fig. 1). Additionally, STRT-seq is another method based on UMI that uses streptavidin beads, which makes the subsequent library construction, quality control, and other processes more accurate and convenient [48, 51].

The data processing methods for scRNA-seq are also very important. Since the amount of RNA from a single cell is very limited, scRNA-seq depends largely on amplification. Such large amounts of RNA amplification causes random dropout events, thus inducing a large number of zero counts in the expression matrix. Therefore, the analysis of scRNA-seq data needs more professional normalization methods. Some of the commonly used data processing methods include Seurat, Monocle, Scanpy, Linnorm. These data processing algorithms can be used for data quality control, gene expression standardization, and dimensionality reduction clustering by different methods. Following this, different algorithm language packages can be selected for detailed functional data analysis, such as marker gene identification, cell-type identification, subgroup analysis, and pseudotemporal ordering analysis [52].

Among them, Seurat is a toolkit for quality control, analysis, and exploration of scRNA-seq data. The purpose of this algorithm is to enable users to identify and explain the heterogeneous sources of single-cell transcriptome sequencing data, and integrate different types of single-cell data for subgroup analysis, marker gene identification and other data analysis and processing [53].Unlike Seurat, Monocle is a novel unsupervised algorithm. It orders cells by progress through differentiation that dramatically increases temporal resolution of expression measurements in a model of skeletal muscle differentiation [54]. This reordering unmasks switch-like changes in the expression of key regulatory factors, reveals sequentially organized waves of gene regulation, and exposes novel regulators of cell differentiation. Scanpy is an extensible toolkit for single-cell gene expression data analysis. It includes data preprocessing, result visualization, clustering analysis, pseudo-time and trajectory inference, differential expression testing and gene regulatory network simulation. It is based on Python and it can effectively process more than one million units of data sets, and is one of the commonly used scRNA-seq algorithms [55]. In addition, there are many other data processing methods, such as Linnorm [56], NODES, SAMstrt, SCnorm, scran, DESeq and TMM, which can be selected according to requirements.

At present, mainstream commercial platforms for scRNA-seq include 10X Genomics and BD Rhapsody. The sample processing time of both platforms is within 30 min, and the entire process is integrated, including library construction, sequencing and data analysis [57].

Single-cell transcriptomics has been combined with many fields to develop highly flexible and comprehensive sequencing technology. CITE-seq is a new sequencing technology that combines transcriptomics with cell phenotype research, and simultaneously conducts single-cell transcriptome sequencing and cell epitope protein indexing. This technology makes up for the limitation of conventional high-throughput single-cell sequencing methods which do not provide cell phenotype information, such as protein expression level of cell surface markers. Oligonucleotide-labeled antibodies are used in this method. The oligonucleotides-containing barcodes are linked to different cell surface protein antibodies by streptomycin biotin affinity reaction. Following antibody binding, cell lysis, mRNA reverse transcription, amplification, library construction, and sequencing, the expression levels of cell proteinsare is determined by antibody-derived tags and the RNA expression of the gene is expressed by sequencing reads. Therefore, CITE-Seq effectively integrates two assays into a single cell sequencingreaction. More importantly, the two libraries are generated separately, so they can be provided separately, and the relative proportion can be adjusted to obtain the most accurate sequencing results [58]. CROP-seq is another innovative application of scRNA-seq, which combines CRISPR screening with scRNA-seq into a broadly applicable workflow. It mainly includes four parts: (1) introducing a guideRNA (gRNA) vector so that gRNA can be directly detected in single-cell transcriptome data; (2) high-throughput analysis of scRNA-seq; (3) a computational pipeline for assignment of single-cell to gRNAs; (4) analysis of single cell transcriptome data induced by gRNA. CROP-seq directly links gRNA expression to transcriptome responses in thousands of individual cells. CROP-seq enables to pool CRISPR screening with single-cell transcriptome resolution, which will facilitate high-throughput functional dissection of complex regulatory mechanisms and heterogeneous cell populations [59, 60].

## Analysis of tumor heterogeneity and development by scRNA-seq

Although it is recognized that cancer is a complex disease affected by many factors, the most widely accepted genetic theory is that tumorigenesis results from somatic mutation accumulation, i.e., tumors are formed by the accumulation of somatic mutations through evolutionary processes [27, 61]. These mutations occur randomly in different genomic regions, a few of which may lead to the malignant transformation of normal somatic cells [62]. Somatic mutations mainly include gene mutation heterogeneity (base pair replacement, insertion, and deletion) and genomic instability (chromosomal instability, chromosome rearrangement, copy number variations and microsatellite instability).Through next-generation sequencing (NGS), it was found that the occurrence of many tumors, such as breast cancer [63, 64], hepatoma [65, 66]and lung cancer, were related to the mutation of oncogenes.

**Fig. 2:**
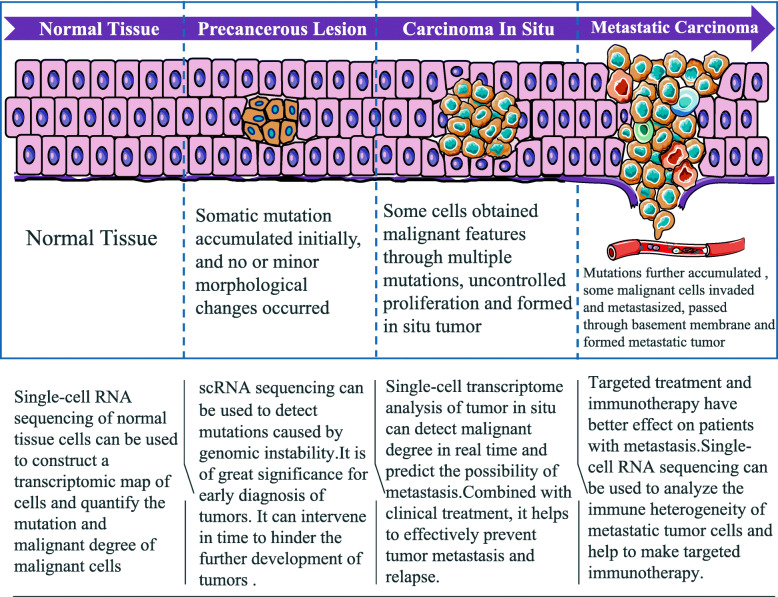
Application of scRNA-seq in tumorigenesis In the process of normal tissues developing from precancerous lesions and carcinoma in situ to metastatic carcinoma, scRNA-seq plays an important role in tumor prevention, diagnosis and treatment

As NGS, scRNA-seq, is used to study the genetic and molecular characteristics of various stages of tumor development(Fig. 2). Therefore, it plays an important role in the process of tumor development from precancerous lesions to invasive tumors, and even metastases [67]. In the case of pancreatic ductal adenocarcinoma (PDA), one of the most malignant tumors, the development has a long transformation process, and precancerous lesions may occur several years before evident manifestation. scRNA-seq is performed on pancreatic epithelial cells with pancreatic intraepithelial neoplasia (PanIN) to analyze gene mutations related to proliferation, invasion, and metastasis, and to evaluate the risk of malignant transformation, so as to provide the possibility to curb further tumor development. Additionally, scRNA-seq is likely to quantify the transcription status of the pancreatic cancer cells and combine the existing clinical data for PDA typing, which is of great significance for clinical targeted therapy [68]. Hosein et al. analyzed the progression of PDA from precancerous lesions and acinar ductal metaplasia (ADM) to malignant invasive tumors in mouse models by scRNA-seq and analyzed the differences in gene expression and heterogeneity of cell composition types. The authors analyzed the cellular heterogeneity in the pathogenesis of PDA and, on the basis of their findings, proposed a new target for targeted treatment of pancreatic cancer [69].

Additionally, several clinical data suggest that tumor metastasis is one of the leading causes of death in patients with cancer and the acquisition of migratory abilities of malignant cells is also an important step in the development of tumor. The metastatic phenotype includes metastasis from the primary tumor location, survival in blood or lymph circulation, invasion of distal tissue, and establishment of distal metastasis [70, 71]. Sridhar et al. compared the gene expression profiles of metastatic and primary tumors in various tumor types and observed the differential expression of some genes. Genetic and transcriptional heterogeneity between primary and metastatic tumors were also noted [72]. These data suggest that analysis of the relationship between metastasis and gene mutation of some tumor cells, may be a new way to control tumor metastasis. scRNA-seq can provide comprehensive information about gene expression and single nucleotide mutations in individual tumor cells and deepen our knowledge in the process of primary tumor metastasis. Chen et al. identified differentially expressed genes in migrating cells by scRNA-seq. With single-cell resolution, they discovered intermediate epithelial-mesenchymal transition (EMT) states and distinct epithelial and mesenchymal subpopulations of migratory cells, thereby indicating that breast cancer cells migrate rapidly while retaining an epithelial state. Migratory cells showed differential profiles for regulators of oxidative stress, mitochondrial morphology, and proteasome. They also identified novel genes correlated with cell migration and outcomes in breast cancer as potential prognostic biomarkers and therapeutic targets to block migratory cells inmetastasis [73].

Kyu-tae et al. elucidated transcriptional heterogeneity in the metastasis of clear cell renal cell carcinoma (ccRCC) by scRNA-seq. The main underlying genetic change in ccRCC is the von Hippel-Lindau (VHL) tumor suppressor gene, whose deregulation leads to changes in tumor invasion, metastasis, and metabolism. scRNA-seq data showed that metastatic renal cell carcinoma (mRCC) had stronger metastasis and invasion abilities. Through data analysis and screening, the authors found that the high expression of EGFR and Src in metastatic renal cell carcinoma could be used as target genes for combined targeted therapy. Moreover, an optimal combination of targeted drugs for mRCC was proposed, which significantly improved therapeutic efficacy in both *in vitro* and *in vivo* models of different cell subsets of mRCC [74].

The application of scRNA-seq technology in the study of tumor heterogeneity is more mature. In addition to the above application in the study of tumorigenesis, scRNA-seq applies in the study of tumor heterogeneity includes cell typing of tumor tissue, and analysis of the characteristic cell state of malignant cells combined with the influence of tumor microenvironment, genetic factors and epigenetic. Neftel et al. performed scRNA-seq in 20 adults and 8 children with glioblastoma (total 24,131 cells), revealing the transcriptional and genetic heterogeneity, and constructed the glioblastoma model and cell lineage. They divided glioblastoma cells into several subclones and identified their specific cell states, such as (I) neural progenitor like (NPC like), (II) oligodendrocyte-like (OPC like), (III) astrocytoid (AC) and (IV) mesenchymal (MES) states. Besides, the authors specified the cell expression program under the influence of cell cycle and microenvironment [75]. Van Galen et al. used scRNA-seq to analyze 30,712 cells from 16 patients with AML and 7,698 cells from five healthy donors and obtained genotyping information of 3,799 cells. The authors analyzed the differentiation trajectory of hematopoietic stem cell (HSC) to various hematopoietic cell types in normal bone marrow samples and divided them into 15 cell groups. At the same time, in tumor cells of patients, gene mutation was evaluated according to single-cell genotyping, then malignant cells were distinguished. Based on marker genes, the malignant cells were further classified into different cell subclones, and the surface marker genes of preferential expression were evaluated, which provided an opportunity for targeted therapy [76].

In past studies, although the research on heterogeneity of cancer evolution and development mainly focused on genetic changes, the latest cancer research data emphasized the contribution of heritable epigenetic modifications to cancer development. In addition to genome-level mutations, epigenetic modification was found to be an important cause of tumor heterogeneity. Environmental and metabolic stimuli can destroy the homeostasis of chromatin, which makes chromatin abnormally tense or loose. Such stimulation can promote the occurrence of tumor in precancerous lesion cells or promote the further development and adaptive acquisition of tumor in malignant cells [77]. Scientists have linked scRNA-seq with epigenetics, making it possible to clearly reveal the epigenetic changes in chromatin tissue in a single cell [78, 79].

For example, Jason D.et al. developed a sequencing technique to analyze the chromatin accessibility of individual cells via assay for transposase-accessible chromatin called as scATAC-seq. They generated DNA accessibility maps from 254 individual GM12878 lymphoblastoid cells and discovered combinations of trans-factors associated with either induction or suppression of cell-to-cell variability. The authors further identified sets of trans-factors associated with cell-type specific accessibility variance. Targeted perturbations of cell cycle or transcription factor signaling evoked stimulus-specific changes in this observed variability. The pattern of accessibility variation in cis across the genome recapitulates chromosome topological domains de novo, linking single-cell accessibility variation to three-dimensional genome organization. Taken together, single-cell analysis of DNA accessibility provides new insight into cellular variation of the “regulome” [78].

Clark et al. developed a combined analysis method, scNMT-seq, which can realize chromatin accessibility, DNA methylation and transcriptomic analysis in a single cell in parallel. Based on previous parallel protocols such as single-cell methylation and transcriptome sequencing (scM&T-seq), the authors performed physical separation of DNA and RNA prior to a bisulfite conversion step and the transcriptome of the cell was profiled using a conventional Smartseq2 protocol. Meanwhile, nucleosome occupancy and methylation sequencing (OMe-Seq) were used to measure chromatin accessibility together with DNA methylation. scNMT-seq can be applied to dissect the dynamics of epigenome interactions during a developmental trajectory and will greatly expand our ability to investigate relationships between the epigenome and transcriptome in heterogeneous cell types, as well as across developmental and other cell fate transitions [80].

In addition, the development and application of single cell RNA sequencing technology supports the cancer stem cell theory. Cancer stem cell (CSC) is an important factor in the cause of tumor heterogeneity. The CSC model provides one explanation for the phenotypic and functional heterogeneity among cancer cells in some tumors. In this model, tumor tissue is divided into carcinogenic cancer stem cells and non-carcinogenic cell subsets. CSCs can divide and expand the pool of CSCs and differentiate into heterogeneous non-tumorigenic cancer cell types. CSCs are thought to have the potential to drive tumor growth and promote disease progression, and are associated with distant metastasis and resistance to treatment. CSC theory requires us to rethink the way we diagnose and treat tumors, because our goal must shift from eliminating a large number of rapidly dividing but terminal differentiated tumor components to focusing on a small number of stem cells that promote tumor growth [81, 82].

Fendler et al. developed procedures to isolate CSCs from ccRCCs and analyzed them through expression profiling and single-cell sequencing. Transcriptional profiling and single-cell sequencing showed that Wnt and Notch signaling were activated in these CSCs. The authors used CSCs from the tumors to produce three model systems—non-attached sphere cultures, 3D organoids, and PDX tumors—to overcome the limitations imposed by single model systems. They treated each model with small molecule inhibitors targeting Wnt and Notch at different stages. Xenograft therapy provided further evidence that specific patient populations can benefit from suppression of Wnt and Notch therapy [83]. In addition, The authors found a small subgroup of CSC by single-cell sequencing and determined the degree of heterogeneity in CSC. The above experimental results provide new ideas for tumor drug resistance and clinical application of CSC therapy [84].

Pan et al. identified a CSC population of 1068 cells and used the Monocle method to reveal a pseudotemporal ordering for the similarity of tumor subclusters with developmental lineages. The authors applied the improved pseudotime trajectory axis with a tree-like structure and found that CSCs positioned as a center of the differentiation process sequentially transformed into cancer 1–4 clusters. In addition, authors used t-SNE with RNA velocity analysis as a visual and intuitive way to observe the extent and direction of tumor cell differentiation. The cells in CSC cluster showed the highest degree of malignancy and were regarded as the starting point of each differentiation direction. These CSCs were located at the center of the differentiation process and transformed into primary and metastatic cells of collecting duct renal cell carcinoma (CDRCC) in time and space sequence. The authors also found that CSC-specific marker genes BIRC5, PTTG1, CENPF and CDKN3 were associated with poor prognosis of CDRCC. Finally, authors suggested that PARP, PIGF, HDAC2 and FGFR inhibitors that effectively targeted CSCs might be potential therapeutic strategies for CDRCC [85].

Single cell RNA sequencing has limitations in detecting somatic mutations and tumor heterogeneity. The disadvantages include 3’or 5’ bias in various sequencing methods, low sensitivity of low-abundance transcripts, high level of noise in scRNA-seq data, insufficient sequencing depth at single-cell level, among others [33, 34]. Scientists have proposed new methods to solve these problems. For example,Anna S Nam et al. developed Genotyping of Transcriptomes (GoT), integrating genotyping with high-throughput droplet-based scRNA-seq. It captures somatic genotypes and transcriptomic identities in thousands of single cells from primary cancer specimens at the same time. Building on previous experience for targeted amplification in droplet-based scRNA-seq, GoT overcomes the unique set of challenges presented by somatic mutation genotyping, including low expression levels and large distances from the end of the sequenced transcripts [86]. InferCNV (inferCNV of the Trinity CTAT Project. https://github.com/broadinstitute/inferCNV) is used to explore tumor scRNA-seq data to identify evidence for somatic large-scale chromosomal copy number alterations. This is done by exploring expression intensity of genes across tumors in comparison to a set of reference ‘normal’ cells. InferCNV provides access to several residual expression filters to explore minimizing noise and further reveals the signal supporting copy number alterations (CNAs). To sum up, there are many problems to be solved in the application of single cell RNA sequencing in tumor heterogeneity research; however, scRNA-seq has good development prospects.

## scRNA-seq and immunocyte typing

**Fig. 3:**
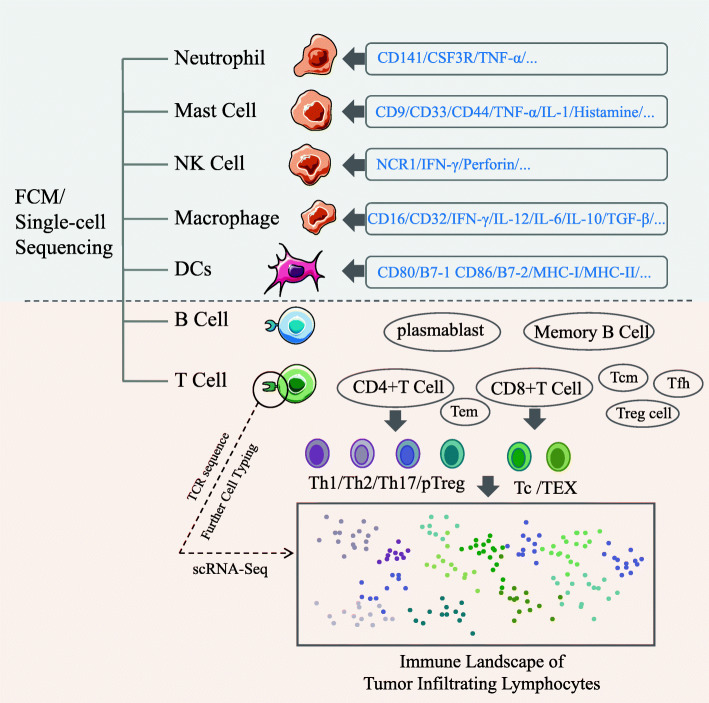
scRNA-seq for immunocyte typing Advantages of scRNA-seq technology to classify the tumor infiltrating T cells, and to provide a detailed landscape of the overall tumor infiltrating immune cells

Tumor heterogeneity includes many aspects, among which immune heterogeneity is an important aspect, which is related to drug resistance and immunotherapy. As Fig. 3 shows, scRNA-seq confirms that tumor tissues contain complex immune components including innate immune cells, such as dendritic cells (DCs), immature dendritic cells (iDCs), activated dendritic cells (aDCs), eosinophils and neutrophils, mast cells, macrophages, natural killer cells (NK; NKCD56 dim cell, NKCD56 bright cell); adaptive immune cells, such as T helper cells Th1 and Th2, regulatory T cells (Treg), CD8 + T cells, central memory T cells (Tcm), memory effector T cells (Tem), T follicular helper cells (Tfh) and γδ T cells, etc [87–90].

The immune heterogeneity is reflected in the degree of immune infiltration and the type of immune cells. There are evident individual differences in patients with different tumor types and developmental stage of the cancer [20]. Melanoma and non-small cell lung cancer (NSCLC) are characterized by high T cell infiltration, while tumor infiltrating lymphocytes (TIL) are rarely observed in immunotherapy insensitive tumors, such as ovarian cancer and prostate cancer [91]. Many studies have shown that the degree of T cell infiltration in tumor tissues is closely related to the survival and immunotherapy of the patients [92]. Generally, CD8^+^ cytotoxic T cells are associated with a longer disease-free survival period. The higher the degree of CD8^+^ T cell infiltration, the better the prognosis [90]. Treg cells have the opposite effect, and the quantity of Treg cells in tumors is inversely related to the sensitivity of immunotherapy [93]. Woosung et al. performed scRNA-seq on tumor tissues of 11 breast cancer patients and showed that the TIL mainly included T lymphocytes, B lymphocytes, and macrophages. Most of the T cells and macrophages showed immunosuppressive characteristics, with T cells displaying an exhausted phenotype and macrophages displaying an M2 phenotype. This suggested that scRNA-seq could find important cell subsets, and might be used as an effective means to develop more effective targeted therapy for tumor types that are not sensitive to immunotherapy, such as triple negative breast cancer [94].

With higher detection accuracy, scRNA-seq can be used to accurately classify tumor cells, and especially identify the distinct forms of immune cells, including helper T cells, cytotoxic T cells and other T cells [95–97](Fig. 3). Zhang et al. obtained 11,138 T cells from patients with colon cancer and systematically studied the tissue distribution, clonal expansion, migration, and developmental transition or differentiation. Analysis of the highly expressed genes and specific T cell receptor (TCR) sequences of the different T cells may further classify the T cells of patients and depict the immune landscape within the tumor [98](Fig. 3). Therefore, scRNA-seq enables the analysis of the specific characteristics of different tumor infiltrating immune cells, thereby designing targeted treatments and improved immunotherapies to achieve better and broader clinical therapeutic effects. Yu Pan et al. identified the subsets of tumor infiltrating immune cells after anti-CD47 treatment by scRNA-seq and revealed that the numbers of CD4 + T cells and CD8 + T cells were significantly increased, whereas that of Treg cells were decreased, all of which enhanced the sensitivity of the tumors to immunotherapy. Here, scRNA-seq technology had a unique advantage compared to the overall sequencing analysis of cell subsets. The experimental data accurately reflected the changes of tumor microenvironment caused by the anti-CD47 treatment, thus confirming that anti-CD47 immunotherapy increased the anti-tumor effect of immune cells and reduced the numbers of macrophages related to immunosuppression. Moreover, it showed that both the innate and adaptive immunity played an important role in anti-CD47 immunotherapy [99].

## scRNA-seq and immune escape

Immune surveillance is one of the most basic functions of the immune system whereby it prevents the occurrence of tumors by recognizing, killing, and eliminating mutant cells in the body. However, recent advancements in science and technology have shown that the immune system can not only protect the host against the tumor development, but can also promote the growth of the tumor by selecting tumor cells with low immunogenicity [100, 101]. To better describe the role of the immune system in the occurrence and development of tumors, the concept of “immunoediting” has been proposed. Through the continuous interactions between tumor cells, immune cells, and the tumor microenvironment, tumor development is divided into three successive stages: elimination, equilibrium, and escape [102]. In the elimination stage, the occurrence of tumors and invasive growth destroys the surrounding tissues, induces inflammatory signals, and thus activates immune cells, eventually leading to tumor cell death. In the equilibrium stage, the host immune system and tumor cells that survived during elimination enter a dynamic balance. The high immune heterogeneity in the tumor enables some malignant cells to adapt and resist the attack from the immune system [102]. In the escape stage, the surviving tumor cells that are insensitive to immune attack by genetic variation begin to proliferate in an uncontrolled way, thereby leading to clinically observable malignant diseases. If this stage is not controlled, it will ultimately lead to the death of the host [101].

**Fig. 4:**
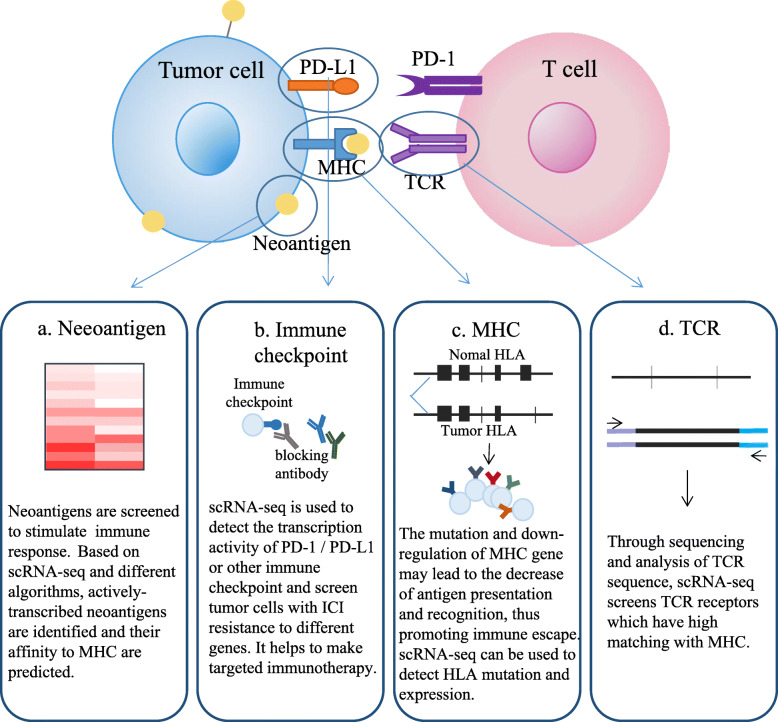
Application of scRNA-seq to evaluate tumor immune escape. **a** scRNA-seq is used to detect and screen for neoantigens. **b** Detection of transcriptional activity at immune checkpoints and evaluation of ICI drug resistance mechanisms by scRNA-seq. **c** scRNA-seq detection of immune escape caused by MHC mutations. **d** Identification of TCR sequences within different T cells by scRNA-seq

Recent studies have focused on the understand of the molecular mechanisms underlying tumor immune escape, and studies have shown that tumors can directly or indirectly suppress anti-tumor immune responses by inhibiting T cell activity. Immune checkpoint inhibitor therapy (ICI) is based on these mechanisms, and it has become a hot spot in the field of tumor research. ICI has been reported to activate the anti-tumor activity of adaptive immune cells such as CD8^+^ T cells [103](Fig. 4). Multiple T cell immune checkpoints have been reported. Among them, immunotherapy targeting two protein antigens, cytotoxic T-lymphocyte-associated protein-4 (CTLA-4) and programmed cell death protein-1 (PD-1), have been extensively studied. It has produced significant clinical effects on melanoma, renal cell carcinoma, NSCLC, and bladder cancer [104–107]. Presently, other immune checkpoints and inhibitory pathways are being studied, such as LAG-3 [108], TIM-3 [109], VISTA [110], and B7-H3 [111].However, due to the high genetic instability of most of malignant cells that have escaped the immune system, the tumor heterogeneity indicates that ICI is not effective for all patients. In certain immunotherapy-sensitive tumors, ICI is only effective on a small number of patients and the effect of heterogeneity on tumor immune response and prognosis of immunotherapy remains difficult to predict [112]. scRNA-seq can be used to analyze the heterogeneity of immune-related genes in tumor tissues, and to design a combination therapy strategy targeted at a variety of tumor antigens to improve the clinical therapeutic effect of immunotherapy [31, 113](Fig. 4). Noemi et al. constructed an immune checkpoint gene network for follicular lymphoma (FL) patients who are insensitive to CTLA-4 and PD-1 treatment. By scRNA-seq of FL and tonsil samples, 11 immune checkpoints including LAG3, HAVCR2 (TIM3), TIGIT, CD27, CD40LG, ICOS, TNFRSF9 (4-1BB), TNFRSF18 (GITR) and TNFRSF4 (OX40R) were selected. With the analysis of the genes that were co-transcribed and co-expressed with these 11 immune checkpoints, the authors found new participants within the immunoregulation process, and constructed a gene network of immune checkpoints in the invasive T cell lineage, which enabled further understand the complex mechanism of tumor immune escape and provided ideas for the further development of immunotherapy [114].

For tumor patients who are not sensitive to anti-CTLA-4 and anti-PD-1/PD-L1 treatment, scRNA-seq can be used to analyze specific tumor tissues and tumor cells and discover the mechanism of drug resistance (Fig. 4). Sangeeta Goswami et al. analyzed ninety-four patients with five different cancer types who received immune checkpoint treatment, including those who were not sensitive to the immune checkpoint treatment. They identified a specific group of CD73hi macrophages in the samples of glioblastoma patients who underwent anti-PD-1 therapy using scRNA-seq. The expression of immunosuppressive genes in the CD73^hi^ cells was enhanced, and the CD73^hi^ cells reduced T cell infiltration in the tumor tissues and reduced the sensitivity of immunotherapy in the patients. Therefore, the authors proposed a combination therapy strategy to target CD73 as well as dual blockade of PD-1 and CTLA-4, which might have a high clinical value [115].

Clinically, some patients with strong immunity develop tumors. In addition to the immuno suppression caused by low T cell infiltration, another important reason for tumor cells to achieve immune escape is the lack of tumor cell immunogenicity [116]. The genetic changes and epigenetic variations of malignant cells, which lead to the abnormal expression of protein and the formation of a variety of tumor antigens, are the principal causes of immunogenicity. Neoantigens can induce anti-tumor immune responses by recognizing neoantigen-specific T cells; therefore, they become new targets for tumor immunotherapy. Since not all mutations can trigger immune responses, that is, they have no immunogenicity, so it is difficult to distinguish the neoantigens with immunogenicity from gene mutation only. The NGS technology first detects the abnormal mutation sites in the tumor cells, and then combines the molecular characteristics of Major Histocompatibility Complex (MHC) to screen the mutant proteins with high binding potential to MHC, which can accurately predict the neoantigens that can be used for the reatment [117–120](Fig. 4). Ma et al. detected the expression of known antigens by scRNA-seq and screened neoantigens that were differentially expressed in lung adenocarcinoma cells. The authors reported several target genes with high specific expression in tumor tissues, and proposed that these could be used as targets in combined immunotherapy for lung adenocarcinoma, such that immunotherapy could better overcome the immune escape and drug resistance [31].

Gubin et al. used the T3 murine methylcholanthrene (MCA)-induced sarcoma line and established T3 tumors in four groups of naive mice, followed by treatment of each group with either control mAb, anti-PD-1, anti-CTLA-4 or anti-PD-1/anti-CTLA-4. scRNA-seq and CyTOF were used to analyze the expression and distribution of infiltrating immune cells, monocytes and macrophages in tumor tissues after different treatments. The results showed that the proportion of CD4 + and CD8 + T cell subsets changed with the development of ICT, which indicated that the activation of antigen-specific CD8 + T cells was the most obvious. At the same time, monocytes/macrophages also underwent significant remodeling after ICT: macrophages in the Mac_s3, Mac_s4 and Mac_s5 clusters increased dramatically upon ICT and achieved maximal levels in the combination ICT group.Mac_s4 clustersare active in inflammatory responses and hypoxia conditions and have strong glycolytic characteristics. Mac_s4 and Mac_s5 clusters showed overlapping characteristics. scRNA-seq and CyTOF reflected the influence of ICT treatment on tumor infiltrating immune cells, which enabled the identification of the best combination of currently available immuno tumor drugs, improved the efficacy of immune checkpoint therapy and identified potential biomarkers, making cancer immunotherapy more effective, specific and safer [121].

In patients with resistance to ICI, this may also be due to mutations in human leukocyte antigen (HLA), an important recognition factor in the immune recognition. It is suggested that genetic mutations of HLA alleles may lead to the failure of checkpoint inhibitors to play their original roles in immune activation and resistance to ICI [122, 123]. Paulson et al. sequenced thousands of cells from two patients with Meckel cell carcinoma (MCC) who had received immunotherapy and had cancer recurrence 18 months later. The results showed that the transcription of HLA was down-regulated in the sequenced samples of the two patients, which further inhibited the activity of tumor infiltrating CD8 + T cells. This finding was consistent with the HLA-mediated immune escape mechanisms proposed in previous studies. Therefore, the design of effective combined immunotherapy strategies toward HLA saved the patients who were not sensitive to general immunotherapy. Moreover, as this HLA-mediated immune escape mechanism may exist in other tumors, this immunotherapy strategy could achieve better therapeutic effects in multiple tumors [124].

## Analysis of tumor intercellular communication by scRNA-seq

Tumors are complex ecosystems defined by the interaction between heterogeneous cell types (including malignant, immune, and stromal cells). The cellular composition of each tumor, and the interaction between these components, may play a key role in the development of cancer [81]. In addition to the high immune heterogeneity of tumor cells, the heterogeneity of other cell types further increases the complexity of tumor tissue. These different types of cells form a complex interaction network through ligand-receptor interaction, paracrine, autocrine, and other intercellular communication modes, which affect the cell morphology, proliferation rate, invasion, and metastasis of tumor. It can change the microenvironment of tumor and affect the therapeutic effect and prognosis of patients [125, 126].

In the past few decades, through the research on the correlation between tumor microenvironments and malignant phenotype, the traditional view of tumor treatment centered on tumor cells has been modified. The interaction between tumor cells and their local microenvironment has provided a new direction for targeted therapy [127]. scRNA-seq can be used to characterize the abundance and functional status of tumor-related cell types, to detect cell-cell communication, to analyze the relationship between cellular interactions in different individuals, and to study the pathophysiological characteristics of tumor microenvironment, so as to predict the development of tumor and prognosis [125]. It also provides new ideas for the application of single-cell genomics in targeted therapy and immunotherapy.

Studies on tumor heterogeneity show that many tumor behaviors rely on cell-cell communication between cancer cells and cancer stromal cells, so as to realize the mutual influence of all kinds of cells and play their physiological roles in coordination. For example, cytokines secreted by tumor cells, such as interleukins, can recruit macrophages, and the secretion of VEGF can stimulate the migration and proliferation of endothelial cells and promote tumor angiogenesis. Zhou JX et al. systematically compared the expression patterns of 2,558 ligand receptor pairs in seven cell types isolated from melanoma (melanoma cells, T cells, B cells, macrophages, NK cells, CAF cells, and endothelial cells) by scRNA-seq. Based on the specific ligand receptor connection, the interaction network between different cell types was established. A large number of detected cell communication signals, such as growth factors, chemokines and matrix proteins, were reported to be closely related to tumor proliferation, metastasis, cell adhesion, angiogenesis, and immunoregulatory processes, thereby revealing that the interaction network was of great significance for tumor development and prognosis [128]. Additionally, Yuan et al. explored the cell types of glioma cells based on a large number of scRNA-seq data and provided relevant data on the autocrine interactions within glioma cells. The gene expression profiles of macrophages and tumor stem cells in gliomas and the interference between these two cell types were also evaluated. It was reported that some specific ligands and receptors in tumor tissues significantly affected the prognosis of patients through different pathways. The autocrine ligand-receptor pairs of 16 glioma growth factors, including Notch1, Dll1, nlgn3, and GDNF, were highly expressed in tumor tissues. These 16 ligand-receptor pairs were involved in tumor invasion, cell adhesion, cytoskeleton synthesis, cell growth, and proliferation, which were closely associated with glioma occurrence. The prognosis model based on ligand receptor interaction could accurately predict the prognosis of glioma patients [129]. Kumar et al. used scRNA-seq data and computational models to study the intercellular communication between ligand and receptor. Moreover, they developed a calculation method to analyze scRNA-seq data and, on the basis of this method, identified the intercellular communication in six homologous mouse tumor models, and further identified the specific interaction in human metastatic melanoma tumor tissues. It was determined that the scores of chemokine CCR1, CCR2, CCR5, and their ligand interactions were the highest, reflecting that macrophages played an important role in tumor intercellular communication and microenvironment regulation [130].

## Conclusions

After several years of development, scRNA-seq has made significant breakthroughs in technological advancement and clinical application. This powerful transcriptome sequencing tool has considerably influenced tumor research and made great contributions to this field. Since the rapid development of scRNA-seq technology, researchers can use this technique to construct transcriptome databases of different tumor cell subpopulations, improve our fundamental understanding of tumor biology, elucidate the mechanisms underlying tumor occurrence and progression, reveal the heterogeneity of cell subsets in the microenvironment of different tumor patients, and analyze the degree of immune cell infiltration and tumor antigen expression.

In the study of tumor pathogenesis, scRNA-seq technology further verified the molecular mechanisms of tumor genesis by analyzing transcriptomics of each cell subgroup, as well as for rare cell clones. scRNA-seq shows that one or more important gene mutations occur in a few somatic cells, which indicate a series of processes such as precancerous lesions, *in situ* tumor formation, metastasis tumor formation, and cancer relapse. Therefore, through scRNA-seq, we can evaluate the risk of tumor susceptibility through changes in expressions of single cells during the early stages of tumor, detect its progress, formulate targeted intervention strategies as early as possible, and prevent subsequent tumor development.

In addition, single cell RNA sequencing has been combined with a variety of omics research to expand its applications in fields such as epigenetics and proteomics. When combined with genomic methylation and chromatin accessibility analysis, scRNA-seq reveals the impact of epigenetic modification on tumor heterogeneity in cancer patients and provides personalized treatment of patients. CITE-Seq and CROP-seq are new technologies that combine single-cell RNA sequencing with cell phenotype research and CRISPR screening, which are of great significance for tumor cell epitope protein index and tumor drug-resistant cell screening.

For a long time, tumor heterogeneity has been an important obstacle in cancer research for the clinical treatment of various tumors. The complex immune heterogeneity contributes in different patients having different responses to treatment. Recently, immune checkpoint inhibitor-based therapy for different T cell antigen receptors has been proposed as it can effectively relieve the inhibition of T cells in tumor tissues. However, due to the immune heterogeneity of tumors, the reported immune checkpoints are not effective in all patients, with most patients showing significant response differences and drug resistance. This suggests that further analysis of single tissues and single cells in immunotherapy is needed to formulate specific immunotherapy strategies. The observed progress in scRNA-seq technology makes it possible to analyze the composition of the immune system at single-cell levels. Based on the recent sequencing data, we can map immune cells and describe the molecular characteristics of the immune system. Moreover, these findings highlight important roles in the discovery of neoantigens, anti-tumor drug resistance, and immune escape.

In April 2020, a collaboration between the Broad institute, Massachusetts Institute of Technology, MSK Cancer Center, and other research institutions proposed and explained the Human Tumor Atlas Network (HTAN). The culmination of knowledge on the basis of data from single-cell sequencing, scRNA-seq, combined with anatomy, histology, spatial information, exome sequence, proteome, epigenome data, metabonomics, and microbiomes, lead to the generation of the three-dimensional (3D) atlases of cancer transformation, which include the stages of precancerous lesions, primary tumors, and metastatic tumors. Through the comparative analysis of these 3D atlases, it is easier to identify tumor heterogeneity and treatment resistance in different patients during different periods, compared to the analysis using simple genomic data. The HTAN program helps to identify new predictive biomarkers and tumor features, as well as to analyze cell types, cell states, and cell interactions during carcinogenesis. Collectively, this information can be used in cancer prevention, treatment improvement, and drug combination strategies to improve the efficiency of clinical treatment and disease prognosis [131]. This multi-disciplinary research strategy can greatly expand the research scope of single-cell RNA sequencing, and may become one of the new development directions in the future.

While the development of scRNA-seq technology has made marked advancements, certain limitations remain. All the known scRNA-seq protocols for eukaryotic cells are limited to detection of mRNAs with poly(A) tails (poly(A) + RNAs). There is a substantial amount of non-polyadenylated RNAs (poly(A)-RNAs) expressed in mammalian cells. Priming through oligo(dT) avoids the preponderance of uninformative ribosomal RNA (rRNA) sequencing reads. However, this approach inevitably precludes the information of other RNA species without the poly(A) tails, such as circRNA and microRNA. In recent years, many studies have shown that non-coding RNAs, such as circRNA and microRNA, play important roles in the development of a variety of tumors and this has become a hot topic in the research of tumor mechanisms [132–137], but the scRNA-seq technology for non-coding RNA remains in a low level. Fan et al. developed a novel single-cell transcriptome profiling method, named single-cell universal poly(A)-independent RNA sequencing (SUPeR-seq), using random primers with fixed anchor sequences to replace the commonly used oligo (dT) primers. SUPeR-seq can detect poly (a) + and poly (a) - RNA in a single-cell, and minimize the effect of rRNA [138]. Generally, scRNA-seq which includes non-coding RNA is still rare, and its application in tumor research is very limited. Therefore, further developments in this field are important for the further use and potential clinical significance of scRNA-seq.

## Data Availability

Not applicable.

## Abbreviations

ADM: Acinar ductal metaplasia
ccRCC: Clear cell renal cell carcinoma
CDRCC: Collecting duct renal cell carcinoma
CIN: Chromosomal instability CNAs Copy number alterations
CTLA-4: Cytotoxic T-lymphocyte-associated protein-4 CSC Cancer stem cell
DC: Dendritic cell
EMT: Epithelial-mesenchymal transition
FACS: Fluorescence-activated cell sorting
FL: Follicular lymphoma
GoT: Genotyping of Transcriptomes
HLA: Human leukocyte antigen
HSC: Hematopoietic stem cell HTAN Human Tumor Atlas Network
ICI: Immune checkpoint therapy
IVT: In vitro transcription
LCM: Laser microdissection
MCC: Meckel cell carcinoma
MHC: Major histocompatibility complex
MMLV: Moloney mouse leukemia virus
mRCC: Metastatic renal cell carcinoma
NK: Natural killer cells
NGS: Next-generation sequencing
NSCLC: Non-small cell lung cancer
PanIN: Pancreatic intraepithelial neoplasia
PCR: Polymerase chain reaction
PD-1: Programmed cell death protein-1
PDA: Pancreatic ductal adenocarcinoma
scRNA-seq: Single cell RNA sequencing
TCR: T cell receptor
TIL: Tumor infiltrating lymphocytes
TSO: Template switching oligonucleotide
UMI: Unique molecular identifier
VHL: Von Hippel-Lindau
